# Apolipoprotein A-I exchange is impaired in metabolic syndrome patients asymptomatic for diabetes and cardiovascular disease

**DOI:** 10.1371/journal.pone.0182217

**Published:** 2017-08-02

**Authors:** Mark S. Borja, Bradley Hammerson, Chongren Tang, Olga V. Savinova, Gregory C. Shearer, Michael N. Oda

**Affiliations:** 1 Children’s Hospital Oakland Research Institute, Oakland, California, United States of America; 2 Department of Medicine, University of Washington, Seattle, Washington, United States of America; 3 Cardiovascular Health Research Center, Sanford Research USD, Sioux Falls, South Dakota, United States of America; 4 Department of Internal Medicine, Sanford School of Medicine, University of South Dakota, Vermillion, South Dakota, United States of America; University of Milano, ITALY

## Abstract

**Objective:**

We tested the hypothesis that HDL-apolipoprotein A-I exchange (HAE), a measure of high-density lipoprotein (HDL) function and a key step in reverse cholesterol transport (RCT), is impaired in metabolic syndrome (MetSyn) patients who are asymptomatic for diabetes and cardiovascular disease. We also compared HAE with cell-based cholesterol efflux capacity (CEC) to address previous reports that CEC is enhanced in MetSyn populations.

**Methods:**

HAE and ABCA1-specific CEC were measured as tests of HDL function in 60 MetSyn patients and 14 normolipidemic control subjects. Predictors of HAE and CEC were evaluated with multiple linear regression modeling using clinical markers of MetSyn and CVD risk.

**Results:**

HAE was significantly reduced in MetSyn patients (49.0 ± 10.9% vs. 61.2 ± 6.1%, *P* < 0.0001), as was ABCA1-specific CEC (10.1 ± 1.6% vs. 12.3 ± 2.0%, *P* < 0.002). Multiple linear regression analysis identified apoA-I concentration as a significant positive predictor of HAE, and MetSyn patients had significantly lower HAE per mg/dL of apoA-I (*P* = 0.004). MetSyn status was a negative predictor of CEC, but triglyceride (TG) was a positive predictor of CEC, with MetSyn patients having higher CEC per mg/dL of TG, but lower overall CEC compared to controls.

**Conclusions:**

MetSyn patients have impaired HAE that contributes to reduced capacity for ABCA1-mediated CEC. MetSyn status is inversely correlated with CEC but positively correlated with TG, which explains the contradictory results from earlier MetSyn studies focused on CEC. HAE and CEC are inhibited in MetSyn patients over a broad range of absolute apoA-I and HDL particle levels, supporting the observation that this patient population bears significant residual cardiovascular disease risk.

## Introduction

Metabolic syndrome (MetSyn) is defined by elevated plasma triglycerides (TG), blood pressure, fasting glucose, and waist circumference. In addition, high-density lipoprotein cholesterol (HDL-C) is reduced [1]. Beyond traditional lipid markers and elevated blood glucose, patients with MetSyn have a substantial residual risk for cardiovascular disease (CVD) [2]. For example, statin-treated patients with low HDL-C or elevated TG have up to 40% greater CVD risk compared to statin-treated patients with normal HDL-C or TG levels [3,4]. Chronic, low-level inflammation, prevalent in MetSyn [5], is associated with a reduction in HDL’s antioxidative capability [6,7]. The ability of HDL to perform reverse cholesterol transport (RCT), another key atheroprotective function [8], may also be compromised by MetSyn-associated factors.

HDL-C is inversely associated with cardiovascular disease (CVD) risk [9], and low HDL-C is a prevalent characteristic of MetSyn dyslipidemia [1]. However, merely raising plasma HDL-C does not necessarily reduce risk for cardiovascular events, as evidenced by recent drug trials of niacin and cholesteryl ester transfer protein (CETP) inhibitors that significantly elevated HDL-C levels but failed to reduce the risk of CVD events in patients [10–13]. Moreover, genetic loci associated with high HDL-C are not associated with a reduced risk of myocardial infarction [14]. A mutation in HDL scavenger receptor SR-BI gene that leads to the reduced hepatic HDL clearance and elevated HDL-C was associated with an increased risk of coronary heart disease [15], further supporting the notion that plasma HDL-C level is not a reliable predictor of CVD risk. The focus has thus shifted to HDL function rather than HDL-C levels to identify the origins of HDL’s atheroprotection.

One of the most frequently utilized measures of HDL function is serum cholesterol efflux capacity (CEC), which measures the ability of HDL to mobilize cholesterol from cell membrane-associated transporters such as ATP binding cassette transporter A1 (ABCA1) in culture cells [16]. ABCA1-mediated CEC measures one of the earliest steps in RCT and exhibits a strong inverse relationship with both incident and prevalent CVD [17–19]. However, studies of CEC in MetSyn cohorts have yielded contradictory results with several reports of enhanced CEC [20–24], and at least one report of impaired CEC [25]. High triglyceride (TG) levels are a prevalent feature of MetSyn and are implicated in artifactually elevating measures of CEC even when HDL-C is low [22,23], making it difficult to assess CVD risk by CEC measurements in hypertriglyceridemic subjects. By employing a measure of HDL function distinct from CEC, we can delineate the influence of MetSyn on HDL function from the potentially confounding influence of elevated TG on CEC measures.

Apolipoprotein A-I (apoA-I) is the major protein component of HDL and is essential for HDL biogenesis and function. A key atheroprotective function of apoA-I is its ability to exchange on and off of HDL particles [26–28]. Lipid-poor / lipid-free apoA-I is the preferred substrate of ATP binding cassette transporter A1 (ABCA1) [26,29,30]. ApoA-I exchange is a critical step to ABCA1-mediated cholesterol efflux and *de novo* HDL biogenesis [8]. ApoA-I undergoes significant conformational change when exchanging between HDL-bound and lipid-free states [28], and this shift in conformation can be reliably quantified by electron paramagnetic resonance (EPR) or fluorescent methods using strategically positioned EPR or fluorescent labels in apoA-I [27,31]. The EPR-based HDL-apoA-I exchange (HAE) assay requires minimal sample manipulation and is highly correlated with CEC in normolipidemic subjects [32]. HAE is inhibited when apoA-I is oxidized *in vitro* and in patients with prevalent CVD [27,31]. However, impaired HAE has not yet been conclusively demonstrated in subjects prior to the development of overt diabetes or symptomatic CVD.

In the present study, we examine a cohort of MetSyn patients to test the hypothesis that HAE is impaired in MetSyn patients, who are asymptomatic for diabetes and CVD. We also examine the relationship between TG, CEC and HAE to determine whether TG exhibits an undue influence on one or both measurements. This study provides, for the first time, evidence that HDL function is compromised due to the loss of HDL’s ability to exchange apoA-I in MetSyn patients, a step of RCT necessary for efficient cellular cholesterol efflux. Furthermore, loss of HDL function prior to development of overt diabetes or symptomatic CVD suggests loss of HAE is a contributor to CVD risk, rather than simply a byproduct of disease onset.

## Materials and methods

### Ethics statement

Human protocols were approved by the institutional review board at the University of South Dakota. Written informed consent was obtained prior to the study from all participants.

### Study subjects

Subjects with MetSyn (n = 60) were recruited and evaluated as described [33]. The study was registered at clinicaltrials.gov (NCT00286234). Inclusion criteria for the MetSyn patients were body mass index (BMI) 25 to 40 kg/m^2^, fasting TG > 140 mg/dL, HDL-C > 10 mg/dL, and the ratio of TG/HDL-C > 3.5. Exclusion criteria were presence of secondary causes of dyslipidemia (hepatic, renal, thyroid, or other endocrine diseases), cardiovascular disease, or diabetes mellitus. Control subjects (n = 14) were recruited to match the age and sex distributions in the MetSyn group, with inclusion criteria as described [34]. Plasma samples were isolated within 1 hour following collection and stored at -80°C before use. Samples were collected between 2007 and 2008 and kept in storage at -80°C without being previously thawed, and HAE measurements in this study were performed in 2012. Sample storage time did not affect measurements of HDL function.

### Lipid and lipoprotein measurements

Plasma lipids and lipoproteins were measured using the Vertical Auto Profile technique (VAP; Atherotech, Birmingham, AL).

### ApoA-I quantitation

ApoA-I levels in subjects were quantified as previously described in Savinova, et al [35]. Briefly, lipoproteins were isolated from EDTA plasma by sequential ultracentrifugation in densities 1.006; 1.063; and 1.21 g/ml corresponding to VLDL, IDL/LDL, and HDL fractions, and stored frozen (−80°C) until analysis. Lipoprotein fractions (4.5 μg of protein) were subjected to gradient SDS-PAGE (4–20% Peptide gels, BioRad, Hercules, CA) and stained with Sypro Orange (Invitrogen, Grand Island, N.Y.). Gels were scanned using Typhoon scanner at 532/555 nm excitation/emission wave lengths and analyzed using Image Quant version 5.0. Intensities of all bands were measured as area under the curve with baseline adjusted manually. The absolute amount of protein in each band was calculated based on its fraction of total protein loaded on the gel (4.5 μg per lane). Protein identification was aided by LC-MS/MS, MALDI-TOF, and comparative 2D electrophoresis. By this method, six classic apolipoproteins: apoA1, A2, B, C2, C3, and E were consistently identified. The majority of apoA-I was detected in HDL and a minor amount was detected in the density fraction corresponding to LDL. Total apoA-I levels in plasma were calculated from these measurements.

### Serum cholesterol efflux capacity measurement

ABCA1-specific CEC measurements were performed in baby hamster kidney (BHK) cells overexpressing human ABCA1 under mifepristone control [16,17,36]. The ABCA1-specific cells were labeled for 24 h at 37°C in DMEM containing [^3^H]cholesterol (1 μCi/mL). Cells were then incubated with or without mifepristone (10 nM) for 24 h, followed by 4 h incubation with apoB-depleted serum (2.8%) supplemented with 0.1% BSA. ABCA1-specific CEC was determined as the difference between cholesterol efflux in cells treated with and without mifepristone. Each sample was analyzed in duplicate and an average is reported.

### HDL-apoA-I exchange measurement

HAE was measured using EPR to quantify the binding of exogenous, spin-labeled apoA-I to HDL in plasma as described in Borja, et al [31]. HAE is measured by directly quantifying the binding of exogenous, spin-labeled, lipid-free apoA-I to HDL, wherein there is a coincident (1:1) displacement of resident apoA-I from HDL particles [27,28]. ApoA-I binding to HDL is quantified by monitoring the center field peak intensity of the EPR spin-label’s spectra, whose intensity is conformation dependent. This signal increases linearly as exogenous apoA-I transitions from a lipid-free to lipid-bound conformation [31]. Briefly, freshly thawed plasma was mixed 1:4 with PBS (20 mM phosphate, 150 mM NaCl, pH 7.4) and 24% w/v PEG 6000 (Sigma) was added to a final concentration of 4% and samples centrifuged at 4°C to remove apoB-containing lipoproteins. The clarified plasma was then mixed with 3 mg/mL spin-labeled apoA-I [37] in a 3:1 ratio and drawn into an EPR-compatible borosilicate capillary tube (VWR).

EPR measurements were performed with a Bruker eScan EPR spectrometer outfitted with temperature controller (Noxygen). Samples were scanned first at 6°C, incubated for 15 minutes at 37°C, and scanned again at 37°C. The baseline spectra of spin-labeled apoA-I in PBS was subtracted from results. Maximum amplitude of spin-labeled apoA-I was determined from spin-labeled apoA-I in a fully lipid-bound conformation. All samples were read in triplicate and averaged. HAE was calculated as described [31].

### Statistical methods

JMP 8.0 (SAS Institute, Cary, NC) and GraphPad Prism 6.0 (GraphPad Software, San Diego, CA) were used to perform statistical analysis. Results are expressed as mean ± SD or as least square mean and 95% confidence intervals where appropriate. Differences between means were determined by performing unpaired, 2-tailed Welch’s *t-*test or one-way ANOVA with multiple comparisons. Associations between different parameters were established by univariate linear regression (Pearson’s r). MetSyn and control subjects were included together in all univariate Pearson correlation calculations. Multiple linear regression modeling used Mallows’ C_p_ to identify predictors of HDL function via the least biased, most parsimonious regression model. Predictors were considered bases on their relationship to the metabolic syndrome. Statistical significance was assumed for *P* < 0.05.

## Results

### Clinical characteristics of study subjects

The clinical characteristics of the study subjects are summarized in **Table 1**. Patients with MetSyn exhibited expected characteristics of metabolic dysregulation with significantly lower HDL-C and apoA-I, and significantly higher LDL-C, TG, BMI and blood glucose compared to healthy control subjects. There were 7 MetSyn patients were on statin therapy and 13 were taking medication to lower blood pressure. The latter did not significantly influence HAE or CEC.

**Table 1:** Clinical characteristics of the study subjects.

	Mean (SD)		Welch p-value
Parameter^a^	Control (n = 14)	MetSyn (n = 60)	
**Demographics**			
Age	45±12	47±10	0.49
Sex F (%F)	5 (36%)	25 (42%)	.
Smoker	.	5 (8%)	.
Anti-hypertensive Med	.	13 (22%)	.
Statin use	.	7 (12%)	.
**Non-Lipid Metabolic Syndrome Factors**			
BMI	23±1	32±4	<0.0001
Systolic BP (mm Hg)	112±9	132±11	<0.0001
Diastolic BP (mm Hg)	69±7	83±7	<0.0001
Glucose (mmol/L)^b^	4.9±0.5	5.6±0.6	0.0002
Insulin (uU/mL)^b^	3.2±1.5	14±8.5	<0.0001
HbA1c (%)	5.4±0.4	5.5±0.5	0.45
HOMA-IR	3.46±0.29	0.69±0.09	<0.0001
**Lipid Factors**			
Triglyceride (mg/dL)^c^	75±24	200±83	<0.0001
LDL-C (mg/dL)	108±26	131±36	0.01
HDL-C (mg/dL)	56±9	42±8	<0.0001
HDL2-C (mg/dL)	13±4	8±3	0.0008
HDL3-C (mg/dL)	43±5	34±6	<0.0001
apoA-I (mg/dL)^d^	116±14	98±19	0.0006

^a^ Previously reported (33, 34) except Insulin, HbA1c, HOMA-IR, HDL2-C, HDL3-C, and TG/HDL-C in the control group.

^b^ N = 58 MetSyn patients

^c^ natural log transformed

^d^ N = 56 MetSyn patients

### HDL function

**Fig 1:**
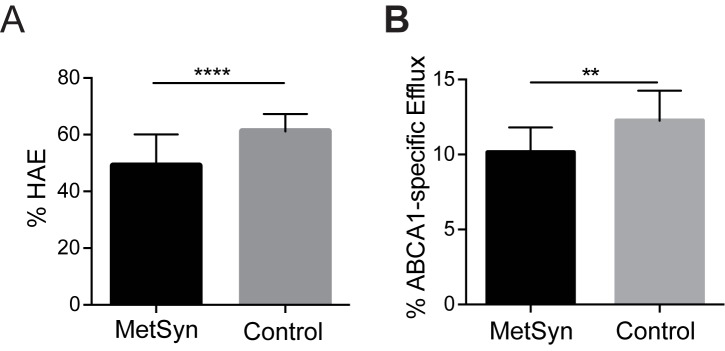
HDL function is impaired in MetSyn patients. (**A**) Average HDL-apoA-I exchange (HAE) and (**B**) ABCA1-specific CEC of MetSyn (n = 60) and healthy control (n = 14) subjects. Error bars represent SD. Statistical significance was determined by performing two-tailed, unpaired *t*-test with Welch’s correction (**** *P* < 0.0001; **P < 0.01).

HDL function was examined using two independent assays: the HAE assay to measure the exchangeability of apoA-I with HDL particles, and the CEC assay to quantify the ability of serum HDL to promote cholesterol efflux via the ABCA1 pathway. MetSyn patients exhibited significantly lower HAE compared to healthy controls (mean ± SD, 49.0 ± 10.9% vs. 61.2 ± 6.1%, *P* < 0.0001, **Fig 1A**). ABCA1-specific CEC was also significantly impaired in the MetSyn group (10.1 ± 1.6% vs. 12.3 ± 2.0%, *P* = 0.002, **Fig 1B**). The inclusion / exclusion of patients treated with statin and / or anti-hypertensives did not significantly alter single- or multivariate model analysis or statistical significance of either HAE or CEC results.

**Fig 2:**
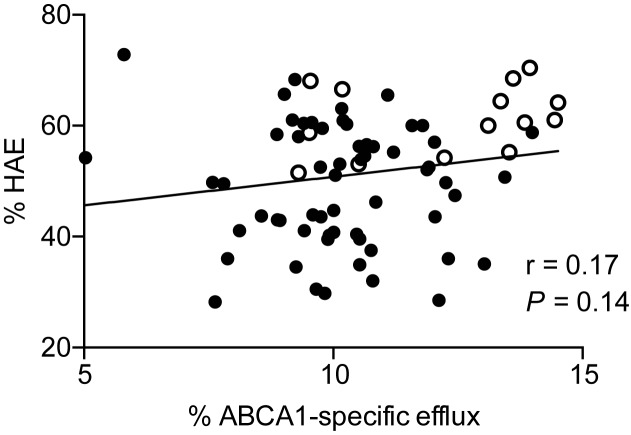
Correlation of ABCA1-specific CEC and HAE. Correlation was determined by linear regression analysis using Pearson’s correlation coefficicent. (●) MetSyn (o) Control. Exclusion of one subject with HAE of 72.8% and ABCA1-specific CEC of 5.8% yields a significant relationship where r = 0.26, *P* = 0.03.

The correlation between HAE and ABCA1-specific CEC was examined by combining MetSyn and control data (**Fig 2**). This analysis revealed a positive but non-significant trend between HAE and ABCA1-specific CEC. However, the absence of significance was driven by a single outlier subject with ABCA1-specific CEC of 5.8%, HAE of 72.8%, apoA-I of 143mg/dL, and z-score = 3.34. When results from this subject are excluded, ABCA1-specific CEC correlates significantly with HAE (r = 0.26, *P* = 0.03).

### Correlation of HAE and ABCA1-specific CEC with lipid and lipoprotein parameters

The correlation coefficients (Pearson’s r) of lipid and lipoprotein factors to HAE and ABCA1-specific CEC are summarized in **Table 2**. MetSyn and control subjects were combined for all correlations. There was a strong positive correlation between HAE and HDL-C (r = 0.62, *P* < 0.0001). The relative levels of HDL2-C and HDL3-C were also positively correlated to HAE to a similar extent (r = 0.54 and r = 0.62, respectively), suggesting that exchange of apoA-I occurs in both HDL subclasses. ABCA1-specific CEC likewise correlated with HDL-C, HDL2-C and HDL3-C (r = 0.39, 0.41 and 0.35, respectively). Neither HAE nor ABCA1-specific CEC correlated with LDL-C. TG levels were significantly elevated in the MetSyn group but did not significantly correlate with HAE or ABCA1-specific CEC. ApoA-I concentration was significantly correlated with HAE (**Table 2**, r = 0.64; *P* < 0.0001), consistent with previous a report [32], and was positively but not significantly associated with ABCA1-specific CEC (**Table 2**, r = 0.24, *P* = 0.056). However, exclusion of the subject noted above resulted in a significant correlation of ABCA1-specific CEC with apoA-I concentration (r = 0.35, *P* = 0.004).

**Table 2:** Pearson correlation of HAE and ABCA1-specific CEC with lipid and lipoprotein parameters.

	HAE		CEC	
Parameter	Pearson *r*	P-value	Pearson *r*	P-value
HDL-C	0.62	<0.0001	0.39	<0.001
HDL2-C	0.54	<0.0001	0.41	<0.001
HDL3-C	0.62	<0.0001	0.35	<0.005
ApoA-I	0.64	<0.0001	0.24	0.056^a^
LDL-C	-0.06	NS	0.02	NS
Triglyceride	-0.14	NS	0.01	NS

NS, not significant

^a^ Exclusion of one subject with apoA-I of 143 mg/dL and ABCA1-specific CEC of 5.8% (z = 3.34, *P* = 0.004) results in r = 0.35, *P* = 0.004.

### Predictors of HAE and ABCA1-specific CEC

To identify clinical parameters that are the best predictors of HAE and ABCA1-specific CEC, we performed multiple linear regression modeling including the following predictors: phenotype (e.g., MetSyn vs. control), age, sex, statin use, anti-hypertensive drug use, TG, LDL-C, HDL-C, HDL2-C, HDL3-C, plasma apoA-I, homeostatic model assessment (HOMA) index, plasma glucose, and diastolic and systolic blood pressure. Since HDL properties are collinear, we controlled for this by evaluating them individually. Thus, we could search for the HDL property (i.e., HDL2-C, HDL3-C, or apoA-I) most predictive of each functional assay without generating unstable models. Mallows’ C_p_ was used to identify the optimal model for explanatory power and parsimony. HAE and CEC are measured using the corresponding tracers (spin-labeled exogenous apoA-I or [^3^H]cholesterol), and their absolute values (as well as differences between groups) are expressed as % of total amount of tracer undergoing HAE or CEC under specified experimental conditions.

**Fig 3:**
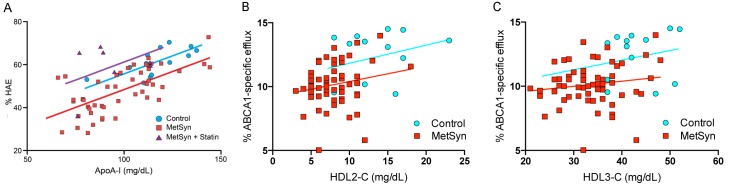
HAE is reduced independent of apoA-I levels in MetSyn patients. Multiple linear regression plot of the predictors of HAE with respect to apoA-I in control, MetSyn, and MetSyn + Statin subjects. Multiple linear regression model of HAE was adjusted for apoA-I, MetSyn status, and statin use. The slope of the regression lines was similar for each group (~0.33), indicating an absence of convergence.

HAE was best predicted by plasma apoA-I concentration (*P* < 0.0001) and statin use (*P* < 0.0001). MetSyn status remained a strong and significant negative predictor of HAE (*P* = 0.004) after adjustment for apoA-I levels. The model explained a large portion of the variance in HAE (r^2^_adj_ = 0.53, **Table 3**). Regardless of subject category, every 1 mg/dL increase in plasma apoA-I corresponded to a 0.33 percentage point (0.23, 0.43 CI; *P* < 0.0001) increase in HAE. MetSyn patients who were not on statin treatment had 7.3 percentage point (-12.2, -2.4 CI; *P* = 0.004) lower HAE compared to control subjects. MetSyn patients who were on statin regimen (n = 7) had 12.8% (6.7, 18.8 CI; *P* < 0.0001) higher HAE compared to those not on statin therapy. Exclusion of the statin-treated MetSyn patients did not decrease the statistical significance of the model and only marginally changed the magnitude of the relationships. The adjusted HAE model plotted with respect to HAE and apoA-I level segregated by subject group, with MetSyn patients exhibiting reduced HAE at every level of apoA-I compared to the control group (**Fig 3).**

**Table 3:** Group differences in HAE^a^.

	%HAE	95% CIs	
Group	LSM	Lower	Upper
Control	61	57	65
MetSyn	48	46	50
MetSyn + statin	61	55	66

r^2^_adj_ = 0.53

^a^ Least square mean (LSM) and 95% confidence intervals (CIs) after adjustment for apoA-I.

ABCA1-specific CEC was best predicted by MetSyn status and proportional TG levels (i.e., natural log(TG)) (r^2^_adj_ = 0.26, **Table 4**). MetSyn status was associated with a 27% lower CEC (-37, -16 CI; *P* < 0.0001) compared to the control group. Natural log(TG) and was positively associated with ABCA1-specific CEC with a 13% (2, 25 CI; *P* = 0.02) higher CEC measure for every natural log(mg/dL) of plasma TG. Statin use was a minor negative predictor, with 12% lower CEC (-22, 0 CI; *P* = 0.05).

**Table 4:** Group differences in ABCA1-specific CEC^a^.

	%ABCA1-specific CEC	95% CIs	
Group	LSM	Lower	Upper
Control	12.7	11.8	13.6
MetSyn	10.7	10.1	11.2

r^2^_adj_ = 0.26

^a^ Least square mean (LSM) and 95% confidence intervals (CIs) after adjustment to natural log(TG), and statin use.

### Relationship of CEC and HAE with TG levels

**Fig 4:**
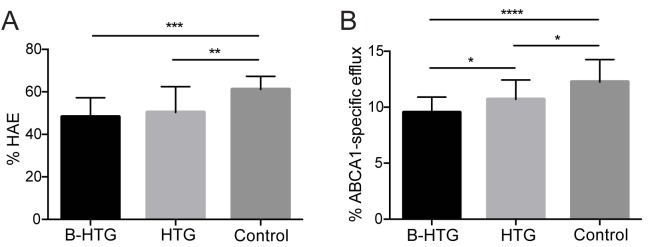
The effect of TG levels on HAE and ABCA-specific CEC. (**A**) HAE (**B**) ABCA1-specific CEC. MetSyn patients were divided into borderline-high TG (B-HTG, n = 29) and high TG (HTG, n = 31) groups and compared to each other and the control group using one-way ANOVA with multiple comparisons (**** *P* < 0.0001, *** *P* < 0.001, ** *P* < 0.01, * *P* < 0.05).

The identification of TG as a strong positive predictor of CEC in the MetSyn group, along with reports in the literature suggesting TG is associated with increased CEC in MetSyn [20–24], warranted further investigation into the effects of TG on both HAE and ABCA1-specific CEC. Current NCEP guidelines classify normal TG levels as <150 mg/dL, while 150–199 mg/dL is borderline-high and ≥200 mg/dL is high TG [38]. The MetSyn patients in the present study were almost equally distributed between borderline-high and high TG (n = 29 and n = 31, respectively). One-way ANOVA with multiple comparisons was used to determine whether HAE and CEC were different between the borderline-high and high TG MetSyn groups, and the control group. Among MetSyn patients, HAE was significantly lower in both the borderline-high and high TG groups relative to the control group (**Fig 4A**, *P* < 0.001 and *P* < 0.01, respectively), with average HAE similar for both MetSyn groups (48.3 ± 1.7% and 50.4 ± 2.1%, respectively). ABCA1-specific CEC in both the borderline high and high TG MetSyn groups was also significantly lower relative to the control group (**Fig 4B**, *P* < 0.0001 and *P* < 0.05, respectively); however, the high TG group had significantly higher CEC compared to the borderline-high TG group (10.7 ± 0.3% vs. 9.5 ± 0.3%, *P* < 0.05), showing that CEC increases with elevated TG levels.

Elevated TG is a marker for insulin resistance and glucose intolerance [39]. However, glucose level was not selected into the multivariate models for HAE or ABCA1-specific CEC. Additionally, models where homeostatic model assessment (HOMA) index score (as a marker for insulin resistance) was forced were evaluated, but resulted in more poorly fitted models, indicating that HAE and ABCA1-specific CEC are independent of insulin resistance and glucose tolerance status.

## Discussion

**Fig 5:**
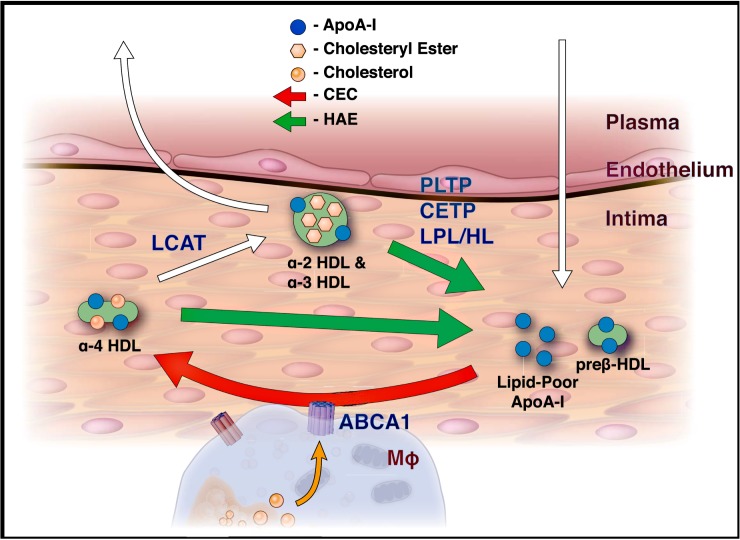
Model of reverse cholesterol transport in the artery intima. White arrows indicate the movement of HDL particles into and out of the intima. Exchange of apoA-I (green arrows) is measured by the HAE assay, with the exchangeable pool of HDL coming primarily from the α-particles. This exchangeable pool of apoA-I is the precursor to preß-1 HDL, the primary substrate for *de* novo HDL biogenesis (red arrow) via ABCA1, residing on cholesterol-loaded macrophages (MΦ).

In this study, we investigated the effect of MetSyn on HDL function, in particular the ability of HDL to exchange apoA-I. *De novo* HDL biogenesis via ABCA1-mediated cholesterol efflux is dependent on the availability of lipid-poor / lipid-free apoA-I [8]. Because apoA-I is not expressed by cells in the intima, the primary source of lipid-poor / lipid-free apoA-I is via exchange from HDL (**Fig 5**). The HAE assay measures the ability of circulating HDL to perform this step. In MetSyn patients, HAE was reduced at every level of apoA-I, demonstrating that loss of HDL function is not merely due to reduced apoA-I or HDL-C levels. Furthermore, the relationship between HAE and apoA-I concentration yielded parallel regression lines for the control and MetSyn groups (**Fig 3**). This pattern of parallel regression lines is consistent with a mechanism of irreversible loss of HAE in MetSyn patients, and because HAE is an essential precursor step to ABCA1-mediated CEC, a large portion of the reduction in CEC is likely attributable to changes in HAE. Further, this is the first study to simultaneously observe impaired ABCA1-mediated CEC and TG-associated enhancement of CEC in MetSyn patients, consistent with the conclusion that dyslipidemic subjects exhibit clinical traits that may unduly influence the CEC assay [23]. This explains why several previous studies examining CEC in MetSyn patients observed elevated CEC, contrary to expectations for a cohort with high CVD-risk.

HAE was significantly reduced in MetSyn patients, and when the adjusted multiple linear regression model of HAE was plotted with respect to apoA-I concentration, MetSyn patients exhibited a lower parallel regression line compared to control subjects (**Fig 3**). The difference in HAE between control subjects and MetSyn patients (not treated with statin) in the adjusted model was highly significant (*P* = 0.004). While apoA-I concentration was a strong predictor of HAE (*P* < 0.0001), MetSyn subjects had lower HAE levels with or without adjustment for apoA-I, indicative of a loss of apoA-I / HDL function in MetSyn patients. A parallel kinetic relationship between HAE and apoA-I concentration was observed when isolated HDL was enzymatically oxidized via myeloperoxidase (MPO) [27], and in acute coronary syndrome (ACS) patients and patients with sickle cell disease in comparison to healthy subjects [31,40]. ACS and sickle cell disease are noteworthy in that both are associated with elevated inflammatory markers [40,41]. In a recent population study of subjects with impaired glucose metabolism, those with MetSyn exhibited both reduced CEC and elevated markers of inflammation [25]. Under conditions of chronic inflammation, HDL is oxidatively modified in the artery intima by the macrophage-derived enzyme MPO [42]. On HDL, apoA-I is the primary target of oxidation by MPO [43], and MPO activity is increased in subjects with MetSyn [44]. Oxidized apoA-I exhibits an irreversible loss of HDL exchangeability [27,31], and reduction in apoA-I’s HDL exchange kinetics results in reduced CEC [27,45]. ApoA-I adduct formation is thus a likely source of HDL dysfunction in MetSyn.

Measuring HDL function in the context of MetSyn has been challenging due to the multifaceted nature of MetSyn dyslipidemia and the array of influences on measures of CEC that arise in this dysregulated physiological state. Previous studies have reported no change of CEC in MetSyn patients [20] or significant increases in CEC in MetSyn patients, proportionate to plasma TG levels [22,23]. Similarly, elevated CEC was observed in insulin-resistant versus insulin-sensitive obese subjects [46]. In contrast, Annema et al. [25] recently reported impaired CEC in MetSyn patients associated with elevated inflammation and no significant correlation with TG levels or impaired glucose metabolism. In the present study, CEC was elevated in proportion to TG levels in MetSyn patients, but when TG was adjusted for in multiple linear regression modeling, MetSyn status was negatively associated with ABCA1-mediated CEC activity, suggesting that the CEC assay identifies HDL dysfunction which is independent of TG levels. It is possible that the decrease in CEC may be related to the loss of HAE stemming from the presence of dysfunctional apoA-I. That HAE and ABCA1-mediated CEC are both impaired in MetSyn patients shows that key early steps of RCT are impaired in MetSyn.

TG exerted a positive effect on the CEC assay, but the HAE assay was not likewise affected. TG enrichment of reconstituted (synthetically derived) HDL exhibited a slightly increased propensity to dissociate apoA-I [47]. However, if this were happening in plasma, CEC and HAE would both be positively influenced in proportion to TG levels. Therefore, a different mechanism is likely involved. In the plasma, TG enrichment leads to an increase in the activity of lipolytic enzymes, particularly hepatic lipase (HL) [48]. When HDL isolated from normolipidemic individuals is enriched with TG *in vitro*, apoA-I dissociation is not observed until the addition of HL [49]. Furthermore, in an *in vivo* study involving rabbits that naturally lack HL, injection of TG-enriched HDL resulted in no change in the dissociation of apoA-I from HDL [50,51]. From these observations, it is likely that TG-associated increases in CEC are due to increased HL activity and not increased exchangeability of apoA-I. It is noteworthy that elevated circulating lipid-free / lipid-poor apoA-I, while associated with increased CEC [16], is also associated with increased CVD risk [52,53]. Thus, the increased levels of circulating lipid-free / lipid-poor apoA-I in the high TG state may not be due to enhanced HAE but rather indicative of impaired HDL maturation or excessive HDL remodeling / lipolysis [54].

Patients with an atherogenic lipid profile (low HDL, high TG) are at increased risk for developing CVD; moreover diabetes patients asymptomatic for CVD with atherogenic dyslipidemia were found to be at higher risk of silent myocardial infarction [55]. The present study excluded patients who were symptomatic for CVD [33], but due to their MetSyn status, these subjects had a high CVD risk profile. The fact that MetSyn patients have impaired HAE at every level of apoA-I, in addition to impaired ABCA1-mediated CEC, suggests loss of these RCT-associated HDL functions contribute to their increased risk for CVD. Recently, Mody et al. reported that CEC measurement when combined with traditional measures of CVD risk (age, sex, total cholesterol, smoking, HDL-C) and emerging risk factors (coronary calcium score, family history, C-reactive protein) significantly improves the prediction of CVD risk [56]. In MetSyn, reduced HDL-C and apoA-I are known markers of elevated risk. Importantly, measurement of the HDL functional parameters, HAE and ABCA1-mediated CEC, revealed that while apoA-I and HDL-C are reduced in MetSyn patients, loss of HAE and CEC functions independent of apoA-I and HDL-C concentration indicate even greater underlying CVD risk. Considering the correlated and complementary nature of HAE and CEC measurements, when combined, these assays could further enhance CVD risk prediction.

Statin treatment had a significant positive effect on HAE, but had a weakly negative association with CEC. There is currently little data on the effect of statin drugs on HAE, with only one small study suggesting simvastatin does not improve HAE in patients with sickle cell disease [40]. Due to the small number of patients on statin therapy in the present study (n = 7), we cannot rule out the possibility that the greater HAE activity observed in statin treated MetSyn patients versus control subjects is coincidental, despite the degree of statistical significance (*P* < 0.0001). However, in a recent study by Khera et al., patients on rosuvastatin had no change in CEC, but apoA-I, HDL-C and HDL particle numbers all increased significantly [57]. Increases in HDL particle numbers along with apoA-I levels would likely increase HAE, and this may be the reason for the high HAE in the statin-treated MetSyn patients in the present study. Additionally, statin treatment can also normalize the plasma lipidome of MetSyn patients [58], which may affect HAE independent of CEC. A larger study will be needed to conclusively determine whether statin treatment restores HDL function in MetSyn patients, and whether this effect is a general effect or restricted to specific statins.

This study has two primary strengths, which are the careful selection of truly healthy controls and MetSyn subjects [33,35] and the use of two unique assays of HDL function. The later made it possible to compare two distinct measures of HDL function relevant to RCT (exchange of apoA-I and efflux of cholesterol from ABCA1) in the context of MetSyn. The primary limitations of the study are the relatively small sample size, the inequality in the number of case and control subjects, and the absence of direct assessement of molecular cuases of HAE and CEC impairment in MetSyn subjects. The reduction in HAE per mg/dL of apoA-I in MetSyn subjects, while spectulative, is consistent with chemical modification / oxidation of apoA-I, as previously reported [27,31,40].

In summary, both HAE and CEC are impaired in MetSyn subjects who are asymptomatic for CVD. The HAE data, in particular, support a model where dysfunctional HDL evolve in MetSyn patients due to irreversible inhibition of the apoA-I exchangeability (HAE) function of HDL, which is indicated by loss of HAE per mg/dL of apoA-I. CEC is also impaired in MetSyn, which becomes particularly apparent in the multiple linear regression model adjusted for TG. Elevated TG promotes increased CEC, most likely through a mechanism of HDL particle destabilization and disassembly [47–51] rather than apoA-I exchange. Our findings support the premise that HDL dysfunction is a source of residual CVD risk in MetSyn patients.
